# New Approaches to Fetal Growth Restriction: The Time for Metabolomics Has Come

**DOI:** 10.1055/s-0039-1692126

**Published:** 2019-06-19

**Authors:** Debora Farias Batista Leite, José Guilherme Cecatti

**Affiliations:** 1 Departament of Obstetrics and Gynecology, Faculdade de Ciências Médicas, Universidade Estadual de Campinas, Campinas, São Paulo, SP, Brazil; 2 Department of Mother and Child Health, Universidade Federal de Pernambuco, Recife, PE, Brazil; 3 Hospital Clínico, Universidade Federal de Pernambuco, Recife, PE, Brazil

**Keywords:** fetal growth restriction, small for gestational age, prediction metabolomics, restrição do crescimento fetal, pequeno para a idade gestacional, previsão metabolômica

## Abstract

Fetal growth restriction (FGR) diagnosis is often made by fetal biometric ultrasound measurements or Doppler evaluation, but most babies are only diagnosed after birth, using the birth weight as a proxy for intrauterine development. The higher risks of neurodevelopmental delay, metabolic syndrome, and cardiovascular illness associated with FGR impose a shift on the focus during pregnancy. New methodological approaches, like metabolomics, can provide novel biomarkers for intrauterine fetal development. Recent evidence on metabolites involved with fetal growth and weight show a consistent role played by lipids (especially fatty acids), amino acids, vitamin D and folic acid. Fetal energy source and metabolism, structural functions, and nervous system functioning need further evaluations in different populations. In the near future, the establishment of a core set of outcomes for FGR studies may improve the identification of the role of each metabolite in its development. Thus, we will concretely progress with the perspective of a translational capacity of metabolomics for this condition.

## Resumo

O diagnóstico da restrição do crescimento fetal (RCF) é frequentemente feito por medidas biométricas ultrassonográficas ou por avaliação pela Dopplervelocimetria, mas, na maioria dos casos, o diagnóstico é apenas pós-natal, usando o peso ao nascimento como um marcador para o desenvolvimento intrauterino. Riscos maiores de atraso do neurodesenvolvimento, síndrome metabólica e doenças cardiovasculares associadas com a RCF impõem uma mudança no foco durante a gestação. Novas abordagens metodológicas, como a metabolômica, podem fornecer novos biomarcadores para o desenvolvimento fetal intrauterino. As evidências recentes sobre os metabolitos envolvidos com o crescimento e peso fetal mostram um papel consistente desempenhado pelos lipídios (especialmente os ácidos graxos), aminoácidos, vitamina D e ácido fólico. A fonte de energia fetal e seu metabolismo, a função estrutural e o funcionamento do sistema nervoso devem ser detalhadamente investigados nos próximos estudos de validação. Em breve, o estabelecimento de um conjunto de desfechos a serem descritos para os estudos de RCF pode melhorar a identificação do papel de cada metabolito no seu desenvolvimento. Assim, iremos progredir no entendimento da RCF numa perspectiva da capacidade translacional da metabolômica para este transtorno.

## Introduction

The impairment of fetal growth has gained major importance over the past few years. There is an increasing body of evidence suggesting that long-term health outcomes could be managed still during pregnancy. Findings among children^1^ and adults^2^ ^3^ ^4^ who were born with a birth weight (BW) below average or who were exposed to maternal undernutrition in utero^5^ ^6^ support the hypothesis of the developmental origins of health and disease (DOHaD). Fetal growth restriction (FGR; or intrauterine growth restriction, IUGR), that is, when the fetus does not reach its ‘optimal’ growth potential, is possibly the underlying condition of future epidemiological burden of noncommunicable chronic diseases (NCDs).

Fetal growth restriction was recognized as a distinct condition in perinatology only in the 1960s,^7^ and it is usually defined by estimated fetal weight (EFW) < 10^th^ centile or fetal abdominal circumference < 10^th^ centile.^8^ Considering the postnatal growth as a development continuum that begins in intrauterine life, BW can be used as a measurement of fetal growth.^9^ Then, FGR (or IUGR) can describe fetal growth impairment of any severity, confirmed at birth or not,^10^ ^11^ ^12^ ^13^ ^14^ while small for gestational age (SGA) neonates can define either FGR or constitutionally small infants.^13^ ^14^ ^15^ However, and unfortunately, there is still little consensus, both from the obstetrics and neonatology standpoints, regarding how clinicians should screen, diagnose and manage these fetuses and newborns. In fact, FGR is responsible for half of the rate of stillbirths,^16^ and the odds of neonatal mortality can be as high as 3.91 (95% confidence interval [95%CI]: 3.21-4.76).^17^ Still, suspicion of fetal growth impairment in pregnancy clearly improves perinatal outcomes.^18^ Clinical factors, ultrasound scan (US) parameters or placental biomarkers have shown modest clues about FGR pathophysiology and management.

Therefore, the development of new strategies for FGR and SGA evaluation is necessary. The postgenomic era is marked by rapid advances in the so-called *omics* sciences, including transcriptomics (the analysis of messenger ribonucleic acid [mRNA]), proteomics (the analysis of proteins) and metabolomics.^19^ The latter is dedicated to studying metabolites, small molecules between 50 and 2,000 Daltons, which represent the complex interaction between each individual and the environment.^19^ ^20^ With metabolomic platforms, it is possible to evaluate endogenous compounds or exposure to contaminants, for instance, and to offer personalized care based on disease phenotype. In pregnancy, it is still an open field to appraise maternal and fetal adaptive responses to the intrauterine environment.

Metabolomic studies have shown maternal metabolic changes during normal pregnancies^21^ ^22^ and how BW is determined;^23^ ^24^ they soon emerged as a promising predictive and diagnostic tool for preeclampsia.^25^ We hypothesize that recent advances in FGR evaluation have at least a similar potential. Therefore, the aims of the present review are to summarize the investigations of FGR with a metabolomic approach, and the future perspectives of translating this knowledge to the bedside practice.

## What are Metabolomics and its Application on Obstetrics?

The first mention of the term ‘metabolome’ occurred in 1998,^26^ and much has been done since then. The metabolome is dynamic by nature, and represents a meaningful simultaneous evaluation of genetic and environmental influences.^27^ As FGR is a heterogeneous syndrome and appears to be a metabolic disorder, both for the mother and the fetus, metabolomics is thought to be the best approach to investigate it.

Nuclear magnetic resonance (NMR) and mass spectrometry (MS) are the most common analytical platforms applied; MS can be coupled with liquid or gas chromatography, for example (Dunn et al^27^ provide a comprehensive review on this issue). Two main types of investigations can be made, with different objectives: untargeted or targeted. The first one, untargeted or ‘metabolic profiling,’ evaluates simultaneously thousands of metabolites in a given sample.^19^ After careful bioinformatics data analysis (principal component analysis [PCA], or partial least squares discriminant analysis [PLS-DA], for example), the peaks must be matched by retention time, accurate mass and spectra. The Human Metabolome Database (www.hmdb.ca) is an example of repository in which the chemical taxonomy (chemical superclass and class, for example) and known biological processes are listed and can be consulted freely.^28^ Metabolic pathways can be checked at the Kyoto Encyclopedia of Genes and Genomes (www.genome.jp/kegg), for example. With untargeted analysis, it is not possible to determine the absolute quantities of compounds, but a relative change between groups. Then, they are generally applied for hypothesis-generating purposes, attempting to comprehend biological processes.^20^ ^27^ In sequence, they should be validated in large-scale studies.^27^

On the other hand, a targeted analysis is hypothesis-driven, that is, devoted to measuring prespecified biomarkers,^19^ ^27^ with acceptable accuracy measurements (sensitivity, specificity, and area under the receiver operating curve [AUC], for example) to differentiate health conditions.^20^ In the latter case, a predictive, diagnostic or prognostic model can be elaborated with as many metabolites as necessary.^20^ Sample preparation will ultimately depend on study design and type of biological sample chosen.^27^ However, it is important to note that biomarkers developed for a given population are only suitable for that population.^20^ Therefore, well-delineated metabolomic research in perinatal medicine has the potential to answer relevant gaps in the clinical practice.

Fetal metabolism depends on the interaction of the fetus with the maternal organism, and it is mediated at the placental level. There probably is a trend towards higher levels of nonessential amino acids (that is, those synthesized by human cells) with increasing gestational age in maternal blood,^21^ while they show downregulated levels in maternal hair.^22^ Some metabolic pathways are suspected to influence BW, such as the carnitine shuttle, de novo fatty acid biosynthesis, 21-carbon (C-21) steroid biosynthesis and metabolism, prostaglandin formation, and glycerophospholipid, glycosphingolipid and tryptophan pathways.^23^ ^24^ Ultimately, these metabolites are involved with energy generation, oxidation of fatty acids,^29^ immune functions,^30^ and cell-membrane organization.^31^ At the same time, environmental exposure to organochlorine compounds, such as phthalate metabolites and perfluorooctanoic acid, are associated to a decrease in BW,^32^ in a sex-specific manner.^33^

It is known that normal pregnancies show metabolic disruption when submitted to any pathological condition, such as fetal chromosomal abnormalities (upregulation of acetone in maternal urine, for example)^34^; hypertensive disorders (downregulation of acetate in maternal urine, for example)^34^; gestational diabetes mellitus (downregulated levels of 2-oxobutyric acid, for example)^22^; restriction of growth, or any combination of those factors.^35^ Changes can be detected in any biological sample, such as blood, urine, hair or even breastmilk.^36^ However, the main difficulty for conducting and interpreting metabolomic studies in reproductive medicine is the significant variety of definitions. For FGR, EFW by US,^11^ ^37^ uterine or umbilical artery blood flow abnormalities,^14^ or BW^10^ ^12^ ^15^ ^38^ ^39^ are all criteria applied to identify these fetuses and newborns. Although not consensual, they represent important FGR phenotypes in the clinical practice. Then, in order to offer a deeper evaluation of the available knowledge, we have kept the definitions applied by each study and present the most recent findings on metabolomic studies with mothers and newborns, near delivery.

## What Metabolomics has Found in Growth-Restricted Fetuses and Newborns

Maternal blood, urine, and hair have been explored for FGR evaluation with metabolomics, as well as amniotic fluid, venous cord blood, and newborn urine. In pregnancy, some studies have associated maternal levels of certain metabolites with BW. Our group has recently suggested a disruption of lipid metabolism in the 2^nd^ trimester of SGA pregnancies (BW < 10^th^ centile).^40^ Untargeted analyses of maternal blood^15^ and hair^22^ ^41^ have provided reliable predictive accuracy that should be validated in different settings. In the third trimester, there is major deposition of fat in fetal tissues and in the brain, which has led to some investigations on maternal fatty acid metabolism. Between 26-28w, linoleic acid levels are positively associated with BW and abdominal adipose tissue volume, while docosahexaenoic acid is related to the proportionality of growth (length/height).^42^ Near delivery, the mother/newborn ratio of medium-chain fatty acids is downregulated in pregnancies affected by IUGR with Doppler abnormalities,^14^ suggesting the increased need of energetic and structural metabolites by these newborns.

Most metabolomic studies with newborns have focused on samples collected near delivery, to get the closest snapshot of fetal metabolism. The findings from neonatal and maternal metabolomic investigations are summarized in Tables 1 and 2.

**Table 1:** Metabolomic studies with newborns

Authors	Country	Type of sample	Time of sampling	Technique	Targeted metabolites	Definition for FGR/ SGA	Participants	Increased metabolites	Decreased metabolites
**Untargeted evaluations**									
Dessì et al (2011)^10^	Italy	Urine	< 24h after birth (before any nutrition)/ again 96h after birth	H-NMR		IUGR: < 10^th^ centile of BW by populational curves suspected in pregnancy	26 IUGR^b^ 30 AGA, preterm births	Creatinine and myo-inositol	
Horgan et al (2011)^15^	Australia	Blood	20' after birth	UPLC-MS		< 10^th^ centile customized birth weight	8 SGA 6 AGA	DG (32:0)	Phenylacetylglutamin, leucyl-leucyl-norleucine, cervonyl-carnitine, (15Z)-tetra-cosenoic acid, hexacosanedioic acid, pentacosenoic acid, cycloheptanecarboxylic acid, hydroxybutyrate, lysoPC (18:2), PC-more than 20 hits, PC, lyso-PC, acetylleucyl-leucyl-norleucinal, lysoPC(16:1), pregnanediol-3-glucuronide, sphinganine 1-phosphate, sphingosine 1-phosphate, pregnanediol-3-glucuronide, 6-hydroxysphingosine
Favretto et al (2012)^11^	Italy	Venous cord blood	Immediately after birth	Metabolic profiling/ LC-HRMS		IUGR^a^: EFW < 10^th^ centile AGA: EFW 10-90^th^ centile – both confirmed after birth by populational curves	22 IUGR 21 AGA; antepartum C-section.	Tryptophan, phenylalanine, glutamate, valine, isoleucine, histidine, proline, methionine, dopamine, uric acid, 5-methylundecenoic acid, L-thyronine, hexadecanedioic acid, (OH)VitD3-3-D-glucopyranoside	
Sanz-Cortés et al (2013)^12^		Venous cord blood	At delivery	H-NMR		IUGR: < 10^th^ centile of BW and Doppler abnormalities	76 IUGR^a^ 55 AGA^d^	Unsaturated lipids, creatine, glutamine	Choline
Dessì et al (2014)^38^	Italy	Urine	1^st^ urine after birth	H-NMR		IUGR: < 10^th^ centile of BW by populational curves suspected in pregnancy	12 IUGR^b^ 17 AGA	Citrate, creatinine, creatine, myo-inositol, betaine/TMAO, glycine	Urea, aromatic, compounds, branched-chain amino acids
Miranda et al (2018)^13^	Spain	Blood	After birth	H-NRM	FGR: EFW <3^rd^ centile or <10^th^ + Doppler abnormalities SGA: EFW and BW <10^th^ without Doppler abnormalities			Fatty acids, formate (SGAxAGA/FGRxAGA) Acetate (FGRxAGA)	
**Targeted evaluations**									
Liu et al (2016)^39^	China	Blood	3-7 days of birth	HPLC-MS	21 amino acids and 55 acylcarnitines	IUGR: < 10^th^ percentile of BW by populational curves	60 IUGR^a^ 60 AGA		Alanine, homocysteine, methionine, ornithine, serine, tyrosine^c^
Visentin et al (2017)^14^	Italy	Venous cord blood	At birth	GC-MS	Medium chain fatty acids	IUGR: EFW < 3^rd^ centile or <10^th^ centile + Doppler abnormalities SGA: EFW and BW < 10^th^ without Doppler abnormalities	11 IUGR 12 AGA 10 SGA	Decanoic and dodecanoic acids (SGA x IUGR) octanoic, decanoic and dodecanoic acids (SGA x AGA)	

Abbreviations: AGA, adequate for gestational age; BW, birth weight; C-section, cesarean section; DG, diphosphatidil glycerol; EFW, estimated fetal weight; FGR, fetal growth restriction; H-NMR, proton nuclear magnetic resonance spectroscopy; HPLC-MS, high performance liquid cromatography - mass spectrometry; IUGR, intrauterine growth restriction; LC-HRMS, liquid chromatography coupled with high-resolution mass spectrometry; PC, phosphocholine; SGA, small for gestational age; TMAO; UPLC-MS, ultra performance liquid chromatography-mass spectrometry.

Notes: ^a^intrauterine growth restriction; ^b^intrauterine growth retardiation. ^c^IUGR < 3^rd^ centile x AGA; 20 early IUGRs, and 56 late IUGRs; metabolites highlighted for both late and early IUGR cases. ^d^55 AGA.

**Table 2:** Targeted metabolomic studies with mothers near delivery

Authors	Country	Type of sample	Time of sampling	Approach	Targeted metabolites	Definition for FGR/ SGA	Participants	Increased metabolites	Decreased metabolites
Visentin et al (2017)^14^	Italy	Blood	After birth	GC-MS	Medium-chain fatty acids	IUGR: EFW < 3^rd^ centile or < 10^th^ centile + Doppler abnormalities. SGA: EFW and BW < 10^th^ centile without Doppler abnormalities	11 IUGR 12 AGA 10 SGA	Hexanoic, octanoic, decanoic and dodecanoic acids (SGA x IUGR) octanoic, decanoic and dodecanoic acids (SGA x AGA)	
Miranda et al (2018)^13^	Spain	Blood	After birth	H-NRM		FGR: EFW < 3^rd^ centile or < 10^th^ centile + Doppler abnormalities SGA: EFW and BW < 10^th^ centile without Doppler abnormalities	27 FGR 25 SGA 28 AGA		Fatty acids, 2-oxoisovaleric acid, citrate (SGA x AGA/ FGR x AGA), alanine (FGR x AGA)

Abbreviations: AGA, adequate for gestational age; BW, birth weight; EFW, estimated fetal weight; FGR, fetal growth restriction; GC-MS, gas chromatography mass spectrometry; H-NMR, proton nuclear magnetic ressonance; IUGR, intrauterine growth restriction; SGA, small for gestational age.

Favretto et al^11^ found 22 metabolites that could differentiate adequate for gestational age (AGA) newborns from newborns with FGR (suspected during pregnancy and confirmed after birth, both EFW and BW < 10^th^ centile). A total of seven were alpha-aminoacids (that is, those involved with protein synthesis), and all compounds were upregulated in FGR newborns. Tryptophan, phenylalanine, and glutamate individually had the best accuracy, reaching 100% of sensitivity (the former two compounds) and at least 85% of specificity (the latter one).^11^ However, in the newborns sampled by Sanz-Cortés et al,^12^ amino acids were only significant in late-onset IUGR (BW < 10^th^ centile with delivery > 35w and normal Doppler evaluation). On the other hand, Liu et al^39^ searched for amino acids and acylcarnitines in neonatal blood. Homocysteine, methionine, tyrosine, alanine, ornithine, and serine showed decreased levels in IUGR < 3^rd^ centile of BW.^39^ While ornithine can be involved with cell proliferation, differentiation and apoptosis,^43^ serine acts as a neurotransmitter of glutamate N-methyl-D-aspartate (NMDA) receptors in brain.^44^ Interestingly, the last two amino acids were upregulated in SGA children without catch-up growth.^45^

In neonatal urine, Dessí et al^10^ ^38^ and Barberini et al^37^ found increased levels of myo-inositol in FGR cases (both EFW and BW < 10^th^ centile). Myo-inositol belongs to the alcohol and polyols chemical subclass.^28^ In adipose cells, it downregulates the release of free fatty acids, and on the ovaries, it mediates glucose uptake and follicle stimulating hormone (FSH) signaling.^46^ Unfortunately, Barberini et al^37^ grouped SGA and large for gestational age (LGA) newborns for a final comparison, but there is some evidence pointing to a higher risk of metabolic events later in life in both groups^2^ ^3^ ^47^ ^48^ of newborns. Interestingly, myo-inositol has been used for the treatment of polycystic ovary syndrome,^46^ which has a known relationship with metabolic syndrome. Thus, more research is needed to elucidate which pathways are affected in SGA and LGA.

Vitamin D has been involved in a multiplicity of biological pathways. It regulates calcium transport through the placenta and parathyroid hormone levels, which may play a role in fetal skeletal development.^49^ Liquid chromatography coupled to MS is the best approach to measure vitamin D levels. Evidence from trials suggest a protective effect of maternal supplementation of vitamin D on BW,^50^ but less is known about its direct impacts on BW or its implications regarding SGA pathogenesis, if there are any. Vitamin D concentration indeed varies according to ethnicity and smoking patterns,^51^ variables already associated with impaired fetal growth. In the 1^st^ trimester, vitamin D levels < 50nmol/L were statistically associated with SGA (BW < 5^th^ centile) infants.^52^ In the 2^nd^ trimester of women with high risk for preeclampsia, vitamin D levels ≥ 75 nmol/L were associated with decreased risk for BW < 10^th^ centile (adjusted risk ratio, [aRR]: 0.46; 95% confidence interval [95%CI]: 0.24-0.87).^53^ However, in low-risk women, levels < 30nmol/L at 15w were not associated with SGA (BW < 10^th^ centile),^54^ even when there were increased parathyroid hormone levels.^55^ These findings suggest that the thresholds of vitamin D that confer either a risk or a protective effect are not the same as those used in the clinical practice to define normal levels in pregnancy. Indeed, apart from the high prevalence of vitamin D deficiency in pregnancy and in cord blood, it appears to have no impact on infant musculoskeletal development at 2y.^51^

This raises the question of whether there is constitutional or truly impaired fetal growth regarding BW. Some researchers have investigated differences between newborns with BW < 10^th^ with or without Doppler abnormalities.^14^ As a matter of fact, metabolic differences are understandable, and perhaps expected, due to fetal blood flow redistribution. Visentin et al^14^ found lower levels of decanoic and dodecanoic acids in FGR (EFW < 3^rd^ centile or < 10^th^ centile plus Doppler alterations) compared to SGA (EFW and BW < 10^th^ without maternal or fetal hemodynamic abnormalities) in both newborns and their mothers. Capric and lauric acids respectively are involved with unsaturated fatty acid biosynthesis, some of them prostaglandin precursors (linoleic acid, for example).^29^ In normal pregnancies, they represent an additional fetal energy source through ketogenesis.^21^ Thus, one could suggest a higher maternal transfer of these non-esterified acids in FGR pregnancies, besides higher fetal use of ketone bodies (energy and structural roles).

In monochorionic twin pregnancies, for instance, amino acid pathways appear to be disrupted in pairs with discordant growth. Cosmi et al^56^ compared FGR (EFW < 10^th^ centile plus abnormal Doppler) infants with AGA infants (EFW > 10^th^ centile, normal Doppler waveforms) from the same index pregnancy. They found downregulated levels of valine, isoleucine (essential branched-chain amino acids), proline and tryptophan (nonessential branched-chain amino acids): proline is part of the composition of collagen, while tryptophan degradation leads to redox cofactors.^30^ At the same time, there was an upregulation of phenylalanine (an essential branched-chain amino acid; precursor of catecholamine neurotransmitters).^28^ These findings need further consideration, but already highlight a metabolic shift in FGR pregnancies.

Ultimately, discriminating the ‘truly restricted fetuses’ from the ‘constitutionally small’ ones may require more than a BW evaluation. From a clinical point of view, it should at least include adverse perinatal outcomes. At the same time, from a translational point of view, this is a great field for advancements with metabolomic studies in a shorter period.

## What Should Be Explored

The World Health Organization (WHO) now recommends iron and folic acid (at least 400mcg) supplementation throughout pregnancy.^57^ Apart from its role in preventing neural tube defects, epidemiological data indicate folate participation on BW. For instance, its depletion is suspected to justify the repeated SGA in case of interpregnancy intervals lower than 23 months.^58^ In fact, a recent systematic review^59^ has pointed that folic acid supplementation before conception decreases the risk of SGA < 10^th^ centile BW (adjusted odds ratio [aOR]: 0.80; 95%CI: 0.71-0.90) or < 5^th^ centile BW (aOR: 0.78; 95%CI: 0.66-0.91).^59^ Additionally, at the nuclei level, folate acts as a methyl donor, and little is known about its involvement with the methylated enriched pathways observed in SGA pregnancies, if there is any.^60^ Therefore, whether folic acid or homocysteine mediate FGR pathogenesis or are only biomarkers of disease merits consideration in further metabolomic researches.

Amino acid supplementation to improve fetal weight is another intriguing relationship. L-arginine is a precursor of nitric oxide, which regulates placental perfusion. Arginine in amniotic fluid is directly correlated with BW, body length and head circumference.^61^ Evidence from small trials show a marked increase in BW (mean difference: 0.41; 95%CI: 0.24-0.58), although the characteristics and follow-up of the participants, as well as the route and duration of arginine supplementation, were heterogeneous.^62^ Arginine is an essential amino acid for infants, and evidence from experimental data suggests its role in inducing protein synthesis that is not dependent on nitric oxide pathways.^63^ Fetal growth restriction placental explants in hypoxic (O_2_ 1%) conditions have half of the metabolites in common with AGA pregnancies under normal oxygen tension (O_2_ 6%), suggesting that hypoxia would play a role in FGR pathogenesis.

As metabolomics is a very sensitive and holistic approach, extra care must be taken regarding sample selection. Evaluating pregnant women or newborns different from those found in the clinical practice will limit the translational potential of this technology. Although guidelines for reporting observational epidemiologic^64^ or metabolomic^65^ studies are available, they do not fulfill the necessary details for translational investigations. Meaningful transfer of the bedside advancements to the clinical practice is a real concern and will be achieved only if researchers and clinicians speak the same language. In the near future, the establishment of a core set of outcomes for FGR studies may organize a description of clinical data and prevent duplicate efforts. Then, we believe that concrete progress with metabolomics will advance faster.

## Conclusion

Metabolomics is a novel and promising area of research in reproductive medicine. Although some results may contradict each other, the maternal and fetal metabolisms are highly dynamic, and may adapt according to several influences. Levels of metabolites in cord blood might represent increased fetal demands or catabolism, for instance. The current available knowledge points to a disruption in fetal energy source and metabolism, structural functions (cell surface membrane, cell proliferation and apoptosis), and nervous system functioning (neurotransmitter pathways). Future validations of metabolomic studies^20^ in different populations will set the ideal thresholds for the clinical practice. Similarly, metabolomic findings may offer clues about FGR prevention (primary up to tertiary) and treatment. At the end, we envision the possible distinction of fetuses that reach ‘optimal growth’ from others that do not.
